# Overhead tank is the potential breeding habitat of *Anopheles stephensi* in an urban transmission setting of Chennai, India

**DOI:** 10.1186/s12936-016-1321-7

**Published:** 2016-05-11

**Authors:** Shalu Thomas, Sangamithra Ravishankaran, Johnson A. Justin, Aswin Asokan, Manu T. Mathai, Neena Valecha, Matthew B. Thomas, Alex Eapen

**Affiliations:** National Institute of Malaria Research (ICMR), IDVC Field Unit, NIE Campus, 2nd Main Road, TNHB, Ayapakkam, Chennai, 600 077 India; Department of Zoology, Madras Christian College, Tambaram, Chennai, 600 059 India; National Institute of Malaria Research (ICMR), Sector 8, Dwarka, New Delhi, 110 077 India; Department of Entomology, The Pennsylvania State University, University Park, PA 16802 USA

**Keywords:** Vector control, *Anopheles stephensi*, Urban malaria, Chennai

## Abstract

**Background:**

Wells and overhead tanks (OHT) are the major breeding sources of the local malaria vector, *Anopheles stephensi* in the Indian city of Chennai; they play a significant role in vector breeding, and transmission of urban malaria. Many other man-made breeding habitats, such as cemented cisterns/containers, barrels or drums, sumps or underground tanks, and plastic pots/containers are maintained to supplement water needs, temporarily resulting in enhanced mosquito/vector breeding. Correlating breeding habitats with immature vector abundance is important in effective planning to strengthen operational execution of vector control measures.

**Methods:**

A year-long, weekly study was conducted in Chennai to inspect available clear/clean water mosquito breeding habitats. Different breeding features, such as instar-wise, immature density and co-inhabitation with other mosquito species, were analysed. The characteristics of breeding habitats, i.e., type of habitat, water temperature and presence of aquatic organisms, organic matter and green algal remnants on the water surface at the time of inspection, were also studied. Immature density of vector was correlated with presence of other mosquito species, malaria prevalence, habitat characteristics and monthly/seasonal fluctuations. All the data collected from field observations were analysed using standard statistical tools.

**Results:**

When the immature density of breeding habitats was analysed, using one-way ANOVA, it was observed that the density did not change in a significant way either across seasons or months. OHTs contributed significantly to the immature population when compared to wells and other breeding habitats of the study site. The habitat positivity of wells and OHTs was significantly associated with the presence of aquatic organisms, organic matter and algal remnants. Significant correlations of malaria prevalence with monthly immature density, as well as number of breeding habitats with immature vector mosquitoes, were also observed.

**Conclusions:**

The findings that OHTs showed fairly high and consistent immature density of *An. stephensi* irrespective of seasons indicates the potentiality of the breeding habitat in contributing to vector density. The correlation between vector breeding habitats, immature density and malaria prevalence indicates the proximity of these habitats to malaria cases, proving its role in vector abundance and local malaria transmission. The preference of *An. stephensi* to breed in OHTs calls for intensified, appropriate and sustained intervention measures to curtail vector breeding and propagation to shrink malaria to pre-elimination level and beyond.

## Background

In Southeast Asia, the second-most malaria-affected region in the world, India has the highest malaria burden, with an estimated 24 million cases per year [1]. Approximately 95 % of India’s population resides in malaria-endemic areas, although 80 % of cases are confined to areas with only 20 % of the population, largely tribal, hilly and inaccessible regions [2]. The state of Tamil Nadu had 8714 malaria cases in 2014, of which 8377 (96.13 %) were *Plasmodium vivax* and the remaining 337 (3.86 %) *Plasmodium falciparum* [3]. Almost 70 % of malaria cases recorded in Tamil Nadu occur in Chennai with 0.28 million permanent anopheline breeding sources, including wells, open overhead tanks (OHTs) and sumps [4].

The National Vector Borne Disease Control Programme (NVBDCP) of India has framed technical guidelines and policies for a malaria control programme. Chennai city was brought under the centrally sponsored Urban Malaria Scheme (UMS) in 1973. In spite of efforts over the past four decades, the present system is unlikely to eliminate malaria due to the rapid rate of urbanization and other socio-ecological factors. The high disease endemicity with prevalence of *P. vivax* in Chennai [5] requires intensive and regular active surveillance to effectively reduce malaria. *Anopheles stephensi*, the vector responsible for urban malaria in Chennai, breeds mainly in clean/clear water containers such as OHTs, wells, cisterns, barrels or drums, sumps (underground tanks), roof gutters, curing pits in construction sites, fountains, and ornamental tanks. The present study aimed to find potential breeding habitats and assess their role in contributing vector density in a highly malarious area of Besant Nagar in Chennai.

## Methods

### Selection of field site and sampling

**Fig. 1:**
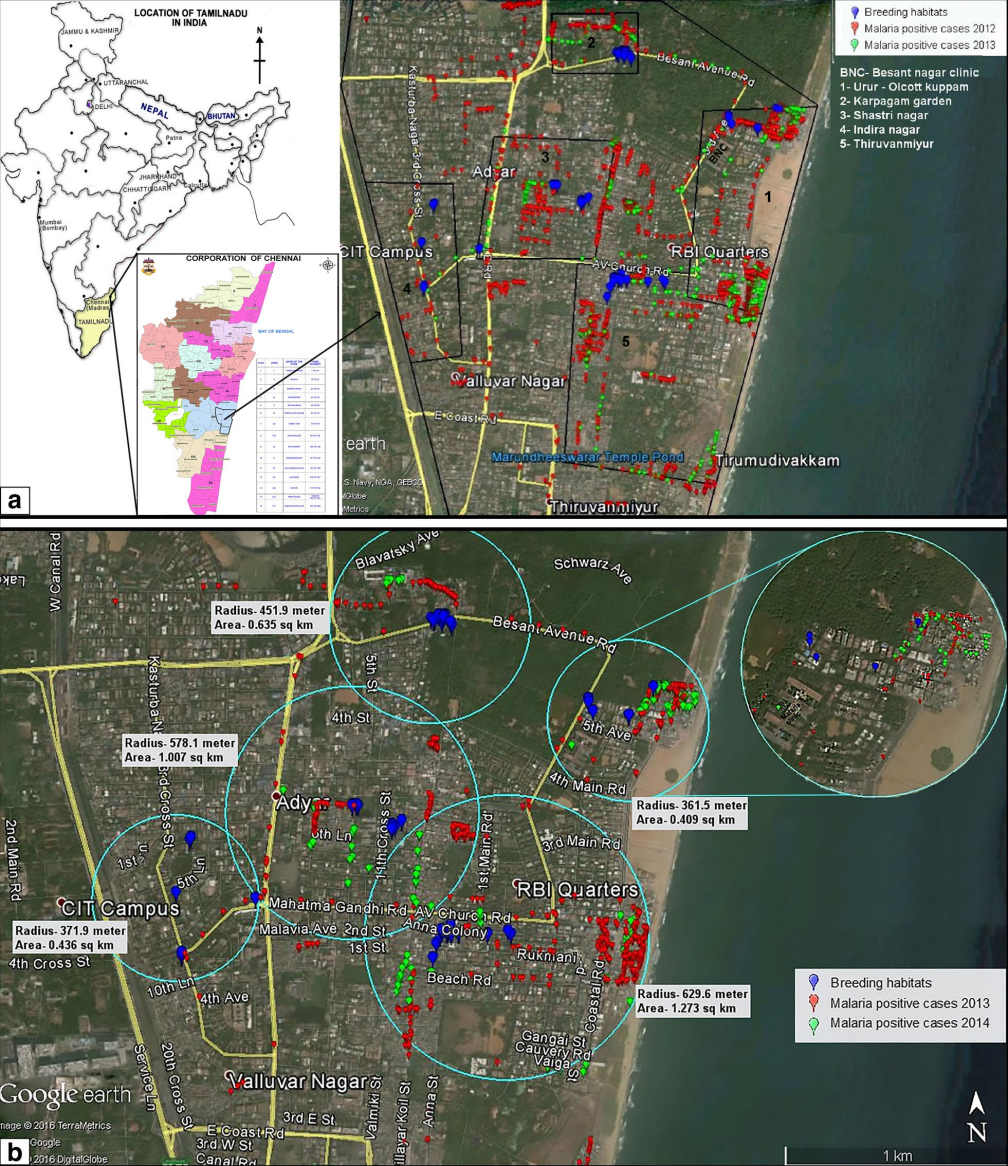
**a** GPS-plotted locations of habitats sampled during the immature vector survey in the study site, together with distribution of the malaria cases recorded at the local malaria clinic during 2012 and 2013. **b** Malaria prevalence and the proximity of breeding habitats in surveyed clusters

The study site, Besant Nagar (13.0002˚N, 80.2668˚E) is a residential area with slums adjacent to the seashore in the southeastern part of Chennai; it is distinctly characterized by its meso-endemic perennial transmission of malaria, predominantly *P. vivax*, by the Asiatic urban malaria vector, *An. stephensi* [6]. The malaria-receptive area of the clinic located at the Regional Office for Health and Family Welfare (ROH&FW), Besant Nagar, Chennai was selected based on malaria prevalence data from 2006 to 2012 and its potential anopheline breeding sources. Malaria cases of 2012 were plotted with the help of global positioning system (GPS) (Garmin—Version 2.40) to identify the major transmission pockets. According to malaria prevalence history and a preliminary study to identify the potential anopheline breeding habitats, the study site was divided into five malarious clusters: Karpagam Garden, Indira Nagar, Urur-Olcott Kuppam, Shastri Nagar, and Thiruvanmiyur. Sites for immature vector collection were selected in and around these clusters (Fig. 1a). The 12-month investigation (April 2013–March 2014) was designed as an annual longitudinal survey to understand the breeding preference and seasonal pattern of *An. stephensi* within this transmission environment. Malaria prevalence and the proximity of breeding habitats in clusters recognized during the study period have been elaborated in Fig. 1b.

### Immature collection

Immature anophelines were collected from wells, OHTs and other clear water storage containers present in each cluster. Damaged/dilapidated OHTs were not considered for longitudinal survey as they had neither water nor any larvicide treatment. Collections were carried out on a weekly basis from April 2013 to March 2014. Immature mosquitoes were sampled using standardized techniques [7–9]. Wells were sampled using ‘well nets’ (conical drop nets 20 cm in diameter and 10 cm deep, that were lowered into the well on a string), while OHTs were sampled using ladles with a volume of 250 ml. In both cases, individual habitats were sampled with four dips per sampling occasion, with immature density of each habitat scored as the number of immatures per dip (i.e., total number of larvae and pupae collected/number of dips taken). Ten sentinel and ten random sites were surveyed every week for each type of habitat. Sentinel sites were selected OHTs and wells that had easy accessibility and with continuous storage of water in order to find out the breeding pattern. Random sites included any accessible well or OHT, with storage of clear water observed during the weekly surveys in the study site. In addition to wells and OHTs, samples were collected from other temporary water storage containers (barrels or drums, sumps or underground tanks, cemented containers, plastic pots/containers, curing pits on construction sites) on inspection, but these habitats were highly ephemeral due to regular consumption and replenishment to adequately low storage capacity. Moreover, the number of such habitats was low due to the frequent water supply in the area.

The survey was carried out in 974 wells and 960 OHTs during the study period. A total of 168 other water storage containers were also sampled. The breeding habitats were visually observed [10–12] to determine the presence of aquatic organisms, organic matter (any decaying biological remnants), and algae (green algal remnants). As anopheline larvae rest on the water surface and are surface feeders, samples were made from the top/surface layer of a water body, manually and thoroughly inspected [13]. Immature density of *Anopheles*, *Aedes* and *Culex* species (if co-inhabiting with anophelines) were also recorded. Sampling was carried out mostly from 09.00 to 12.30 h. Water temperature of the habitats during the time of sampling was also recorded. December–February corresponded to the winter season with comparatively low mean ambient temperatures of 24–33 °C; March–May is the hottest period with temperatures of 26–42 °C; June–November include pre-monsoon and monsoon periods, with intermediate temperatures of 25–39 °C. The atmospheric temperature profile was calculated based on a seasonal study carried out using Hobo data loggers (unpublished data).

The anopheline samples were all categorized and enumerated based on their different immature stages. They were later maintained in the laboratory under controlled conditions (27 ± 2 °C and 70–80 % relative humidity), and fed with dog biscuit and yeast powder mixed in the ratio of 3:1 [14]. The larvae were maintained in enamel/plastic trays (400 larvae per tray). Late or old instars (third and fourth) and pupae were isolated and maintained in separate trays. The emerged adults were identified to species level following standard identification keys [15, 16].

Relative breeding index (RBI) was calculated to provide a comparative metric of habitat suitability by dividing the number of habitats positive for *An. stephensi* by the total number of habitats positive for any mosquito breeding [17]. The immature density was calculated genus/species-wise to find out their relative abundance in different breeding habitats. Statistical analyses (Correlations, ANOVA, T tests, Chi square) were conducted using IBM SPSS statistics software version 21. Institutional ethical clearance of the project was obtained on 20 July, 2010 (ECR/NIMR/EC/2010/100).

## Results

### Correlation between malaria prevalence and breeding habitats

Pearson correlation was executed to find out correlation between malaria prevalence and various parameters (immature density, habitats with anopheline breeding) of breeding habitats. When the number of malaria cases was correlated with immature density, month and cluster-wise, Urur-Olcott Kuppam cluster showed significant correlation (r = 0.764; p < 0.05). When the malaria cases were correlated with number of breeding habitats, cluster-wise, it was highly significant (r = 0.960; p < 0.05). When the number of malaria cases was correlated with number of habitats with presence of vector immatures, month and cluster-wise, significant correlation was found in both Urur-Olcott Kuppam cluster (r = 0.592; p < 0.05) and Shastri Nagar cluster (r = 0.671; p < 0.05). The proximity between breeding habitats and malaria cases within the clusters ranged from 6.22 to 690.77 m.

### Pattern of mosquito breeding and habitat preference

**Fig. 2:**
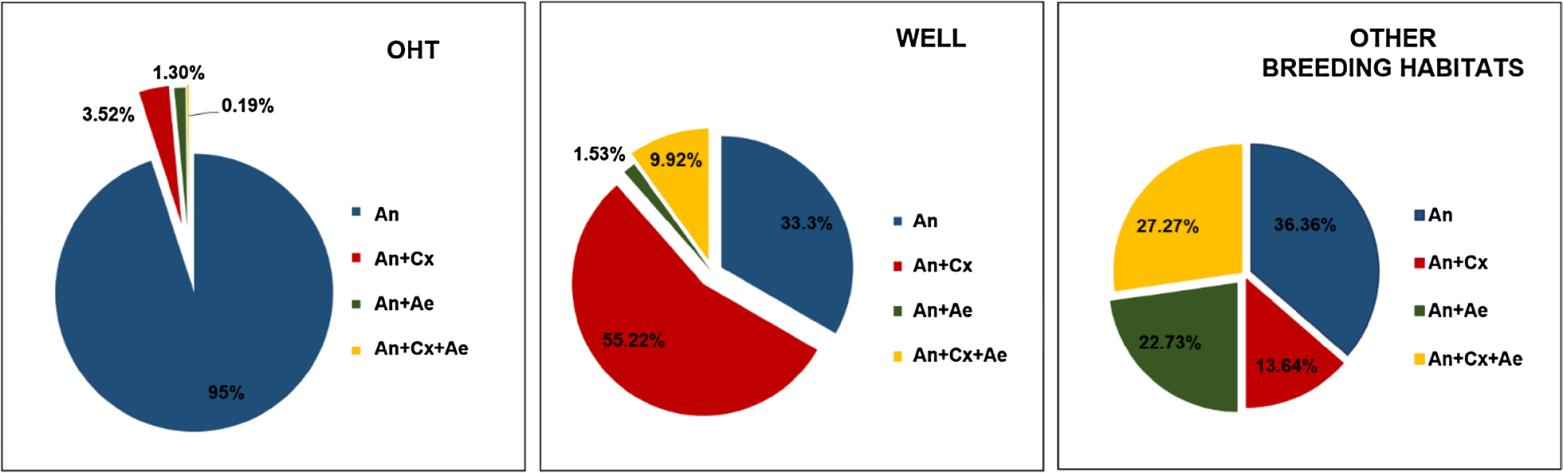
Percentage composition of immature density of *Anopheles stephensi* in association with other mosquito vectors in overhead tanks (OHTs), wells and other breeding habitats

Immature collection revealed that OHTs were the preferred and potential breeding habitat of *An. stephensi*. A total of 37,948 anopheline immatures were collected, out of which, 29,824 (78.61 %) were collected from OHTs, 5296 (13.96 %) from wells, and the remaining 2828 (7.45 %) from other water storage containers. Further, 66.67 % of wells and 63.64 % of other breeding habitats with *An. stephensi* breeding were observed to co-inhabit with either *Culex* or *Aedes* species. However, co-inhabitation of anophelines with other mosquito species in OHTs was rare (5 %). Varied patterns of association and co-inhabitation of different mosquito species in wells, OHTs and other water storage containers are represented in Fig. 2. The larvae and pupae collected from the field survey were visibly healthy and active, and mortality during the process of adult emergence was negligible (<10 %). Rare presence of other anopheline species such as *Anopheles vagus*, *Anopheles subpictus* and *Anopheles barbirostris* (0.04 % of the total collected anopheline larvae) were observed on adult mosquito emergence.

### Seasonal fluctuations of immature density and percentage habitat positivity

**Fig. 3:**
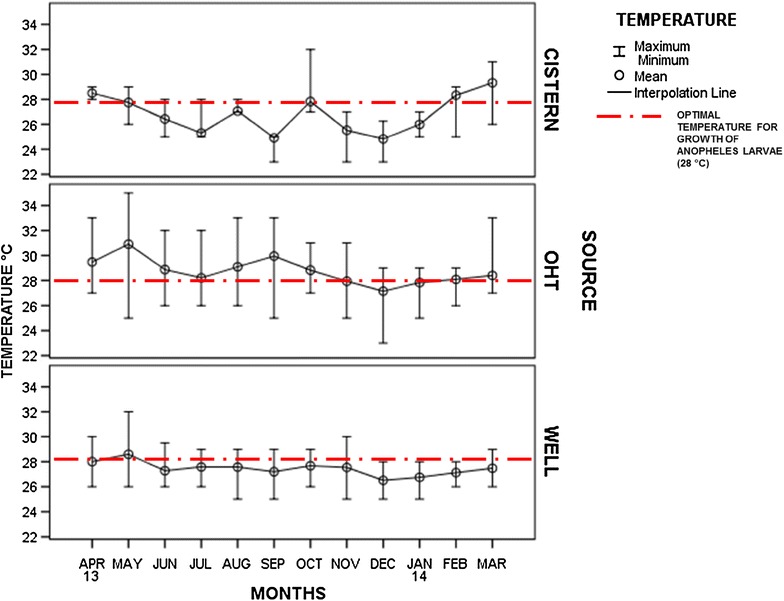
Ambient temperature profile of different breeding habitats and the optimal temperature (28 °C) for the growth of *Anopheles* larvae

OHTs exhibited maximum immature density during April to October whereas wells were most productive during April, May, June, and July. Percentage habitat positivity for OHTs ranged from 48 % (December) to 67.5 % (October) whereas for wells, it ranged from 23.17 % (March) to 69.74 % (June). RBI or abundance of anopheline breeding habitats was high in OHTs ranging from 0.84 (December) to 1 (February, March, October) unlike wells, indicating that OHTs are the preferred ovipositional site for *An. stephensi*. RBI of OHTs was, by and large, constant with continuous breeding throughout the study period. However, in wells RBI ranged from 0.44 in October to 0.81 in August (Table 1). RBI of other breeding habitats varied from 0.00 in January, and 1.0 during February, March and April. The immature densities calculated in general, as well as for anophelines alone, are represented in Table 1. Average water temperature of OHTs recorded at the time of sampling was found to be lowest during December (27.16 °C) and highest in May (30.92 °C). It was surprising to observe that OHTs with water temperature as high as 35 °C (May 2013) were found with *An. stephensi* breeding [18–20]. Similarly, wells recorded maximum temperature in May (28.59 °C) and minimum temperature during December (26.58 °C). For other breeding habitats, the maximum temperature was recorded during March (30 °C) and minimum during December (24.82 °C). Ambient temperature profile of different breeding habitats with the optimal temperature (28 °C) for the growth of *Anopheles* larvae [21] was observed to vary (Fig. 3).

**Table 1:** Immature density and relative breeding index (RBI) of *Anopheles stephensi* in overhead tanks (OHTs), wells and other breeding habitats

Month/year	OHT					Well					Other breeding habitats^b^				
	No. surveyed	% positivity	Immature density^a^	*Anopheles* immature density	RBI	No. surveyed	% positivity	Immature density^a^	*Anopheles* immature density	RBI	No. surveyed	% positivity	Immature density^a^	*Anopheles* immature density	RBI
APR ‘13	95	55.8	9.3	9	1	99	35.4	17.8	2.7	0.5	9	33.3	6.1	1.9	1
MAY ‘13	77	54.6	8.6	8.2	1	76	59.2	15.6	2.5	0.8	5	60	10.3	8.4	0.8
JUN ‘13	80	53.8	5.8	5.3	0.9	76	69.7	13	2.4	0.8	16	75	14.6	5.5	0.9
JUL ‘13	78	56.4	8.5	8.3	0.9	74	56.8	19.8	2.8	0.7	22	36.4	28.1	1.8	0.7
AUG ‘13	81	66.7	10.6	10.5	1	81	59.3	11.6	1.4	0.8	29	17.2	148.9	9.9	1
SEP ‘13	66	59.1	11.7	10.9	0.9	67	34.3	8.4	1.1	0.5	27	7.4	8.4	0.3	0.1
OCT ‘13	80	67.5	9.7	9.5	1	80	28.8	6.2	0.4	0.4	18	11.1	0.1	0.1	0.5
NOV ‘13	74	56.8	5.4	5.4	0.9	79	32.9	2.8	0.4	0.6	18	22.2	34.1	5	0.8
DEC ‘13	75	48	4.4	4.4	0.8	80	28.8	3.2	0.5	0.6	13	15.4	30.8	4	0.4
JAN ‘14	101	50.5	6.4	6.3	0.9	99	31.3	3.8	0.6	0.5	2	0	0	0	0
FEB ‘14	76	55.3	2.9	2.9	1	81	30.9	6.8	1	0.8	6	16.7	6.9	0.5	1
MAR ‘14	77	52	3.8	3.6	1	82	23.2	9	0.5	0.8	3	33.3	38.5	3.6	1

*RBI* relative breeding index (number of habitats positive for *An. stephensi* divided by the total number of habitats positive for any mosquito)

^a^Includes the total density of *Anopheles*, *Culex* and *Aedes* species

^b^Other breeding habitats include underground tanks or sumps, barrels or drums, plastic pots, plastic containers, cemented containers, curing pits in construction site

### Relationship between immature density and environmental parameters

ANOVA was used to compare differences in immature density (immatures/dip) between the seasons, months and habitats. The immature density of breeding habitats for different seasons was not statistically significant. However, when the immature density was averaged per month, the mean immature density was found to be highest during May (4.95 ± 1.25) and lowest during March (1.67 ± 0.30). This difference in monthly mean values was statistically significant (p = 0.01, df = 11, F = 2.267). Also, when the immature density was analysed by habitat, the OHTs were shown to have higher density (5.36 ± 0.38) than wells (1.28 ± 0.11) and this difference was also found to be statistically significant (p < 0.01, df = 1, F = 106.713).

Pearson Chi square analysis was performed to explore the influence of the aquatic organisms, organic matter and algal remnants on the positivity of the habitats. It was found that presence of immature stages of vectors in OHTs were positively associated with the presence of other aquatic organisms (Chi square value = 73.267, p < 0.001, df = 1), organic matter (Chi square value = 94.13, p < 0.001, df = 1) and algae (Chi square value = 69.050, p < 0.001, df = 1). Similar patterns were observed for wells, with *An. stephensi* immatures positively associated with other aquatic organisms (Chi square value = 9.378, p = 0.02, df = 1), organic matter (Chi square value = 6.591, p = 0.01, df = 1) and algal remnants (Chi square value = 4.712, p = 0.030, df = 1).

The density of immature vectors was influenced by aquatic organisms, organic matter and algal remnants. Breeding habitats with aquatic organisms were found to have a higher immature density (3.49 ± 0.22) than those without the aquatic organisms (0.98 ± 0.37) and the difference was statistically significant (p = 0.01, F = 19.87, df = 1932). The breeding habitats with organic matter were found to have a higher immature density (3.57 ± 0.22) than those without them (0.95 ± 0.22), the difference of which was statistically significant (p < 0.001, F = 32.51, df = 1932). Breeding habitats with algal remnants were found to have a higher immature density (3.59 ± 0.23) than those without (1.25 ± 0.27), and the difference was statistically significant (p < 0.001, F = 28.757, df = 1932).

Although the presence of aquatic organisms appeared generally positive for *An. stephensi* immatures, the presence of other mosquitoes had a negative effect. For both breeding habitats combined, *An. stephensi* density was higher when found breeding alone (7.96 ± 0.50), compared with habitats containing either immature *Aedes* spp. (5.02 ± 1.64), *Culex* spp. (4.39 ± 0.64), or all genera combined (3.91 ± 0.84). This difference was also found to be statistically significant (p < 0.001, F = 6.270, df = 3).

## Discussion

Monitoring of adult malaria vector populations can be challenging in densely populated urban areas where there are diverse potential feeding and resting sites and the densities of mosquitoes can be low. In the present study, immature density was chosen instead of adult density, as this accounts for better spatial models for vector monitoring, especially in urban areas [22] and the adult density can be erroneous when the resting nature and preference of vector behaviour changes [6]. In such settings, monitoring of larval densities can provide valuable information on vector density and propagation for understanding local transmission dynamics and to constitute appropriate control measures [22–24].

The data from the immature survey indicated that OHTs are the preferred breeding habitats for *An. stephensi* and represent a potential source to sustain populations throughout the year. Only 2828 immatures collected were from breeding habitats other than OHTs and wells, out of which 2593 immatures (91.70 %) were from a particular cistern kept at a construction site, which suggests that the contribution of these breeding habitats towards immature/adult vector density was practically negligible. When the immature density was analysed across the seasons, it was observed that there was an increase in immature density in OHTs during the monsoon season, whilst wells had the lowest density. This contrasting pattern could possibly be due to heavy rains flushing away and/or mortality of the early or young immatures [25] in wells, unlike in OHTs which tend to be better protected from rains with covers or lids, although not mosquito proofed. It was also observed that a larger proportion of OHTs support breeding of *An. stephensi* compared to wells and other breeding habitats. Whether this is because of differences in abiotic factor such as water quality, or biotic such as the presence of other mosquito species (wells were more often co-inhabited by *Culex* and *Aedes* species) is unclear. Previous research on other mosquito species suggests a functional relationship between the suitability or preference towards a particular oviposition site, the density of potential competitors and the concentration of food resources [26]. In *Anopheles gambiae*, low densities of con-specific larvae were shown to increase oviposition, while high densities, particularly of late instars, deterred oviposition [27]. *Anopheles arabiensis* has been reported to avoid ovipositing where interspecific competitors are present [28]. Relatively little is known about factors determining oviposition behaviour of *An. stephensi* in field settings and this information is really useful to vector control programmes.

In the present study, OHTs showed fairly high and consistent larval density irrespective of seasons, indicating the potentiality of these breeding habitats to invariably contribute to malaria transmission at any time of year. When the immature density of all the mosquito species was calculated, it was found that wells had higher immature density compared to OHTs. However, the anopheline immature density was found to be higher in OHTs. Thus, OHTs were found to be the potential breeding habitat of *An. stephensi* in the study site. Further, the presence of aquatic organisms, organic matter and algae were found to support breeding, as well as immature density in both the breeding habitats in a significant way and could be used as visible or key indicators for vector breeding. Although frequent emptying, with occasional cleaning along with refilling/replenishment of water in OHTs, means the entire source/habitat is renewed every time, it seems to support oviposition through the presence of aquatic organisms or organic matter or algae, as they are reported to act as attractant cues [29]. Wells, which were often found with a self-sustained system of the former factors, support vector breeding, lure other mosquito genera, resulting in intense competition.

Although the basic malaria transmission model indicates a positive correlation between vector density and number of malaria cases, it is well known that small changes in vector density can result in potential changes in the proportion of humans infected, which is more common in low transmission areas such as Besant Nagar compared to those with stable high attack rates [30]. In a highly populated urban area such as Besant Nagar, which covers an area of about 3.5 km (north–south direction) and 2.5 km (east–west direction), the presence of even a few untreated potential habitats could impact transmission drastically since vector dispersion catering to a larger population is quite easy as the flight range of *An. stephensi* is reported to range from 1.8 to 4.5 km [31].

Nevertheless, malaria prevalence depends on many factors: exposure of people to infected bites, immunity level of population, success of malaria positivity detection, attractiveness of vector to a particular individual, personal protection measures, quality of housing, etc. Malaria transmission is influenced by many factors, such as demography (human placement and movement), environmental factors, landscape (vector habitat), socio-economic conditions, which impact malaria transmission in each country and specific locations (foci) [30]. The significant correlation of malaria cases with monthly immature density as well as number of breeding habitats with immatures of vectors clearly points out the role of OHTs as a potential contributor of vector abundance/density which aids in local malaria transmission. Malaria endemicity in the study area (which is under the UMS) invariably reflects a regular release/emergence of adult vector mosquitoes, which is indisputably a result of inappropriately treated habitats, such as OHTs, due to their unapproachability and high immature density compared to other immature habitats.

It is noteworthy that OHTs mainly store chlorinated water supplied by the Chennai Metro Water Supply and Sewage Board (CMWSSB). The temperature data recorded indicated that the water in these OHTs could go to 35 °C in summer (Fig. 3), although these conditions do not seem ideal for mosquito breeding and immature survival [18–20, 32, 33]. Besides, all accessible tanks are programmed to receive routine, weekly larvicidal treatment as part of the UMS. In spite of these factors, *An. stephensi* larvae were collected from around 50–65 % of tanks surveyed throughout the year. These data suggest that treatment of tanks might be quite low or perhaps the dosage of the larvicide may be ineffective in arresting vector density. Many of the OHTs could only be accessed through households and most were not provided with ladders or step-stones, making it difficult for control personnel/staff to undertake anti-larval measures. In addition, replenishment/refilling of tanks could dilute any larvicide that had been applied since water is used continuously for domestic purposes. The data could also indicate issues of tolerance/resistance to the existing dosage of larvicide (Abate-Temephos, an organophosphorous compound) used in the programme [34]. Previous studies indicate both increased [35] or decreased [36] efficacy of organophosphate insecticides when tested under increased larval-rearing temperatures. OHTs that are exposed to direct sunlight may cause degradation of the active ingredient of Temephos, resulting in reduced larval mortality [37].

## Conclusion

Implementation and amendment of the byelaws coupled with political, administrative and societal commitment to mosquito proof OHTs and wells in a phased manner can curtail vector breeding and propagation. The current study suggests greatest emphasis needs to be directed towards arresting vector breeding by ultimately mosquito proofing OHTs as a permanent solution to reduce recurring expenditure on larvicides and manpower. This requires sustained effort and cooperation by the community. Lack of adequate manpower coupled with recurring costs incurred on conventional larvicides is a real burden to the local/national vector control programme. Although larviciding can be used to reduce mosquito vector production, opportunities for environmental management (habitat management or manipulation) should always be sought for long-term measures [9]. Effective implementation of the seven-point action plan [38] where mosquito proofing of potential breeding habitats in every household is mandatory can help in sustained control of vector density and reduce perennial transmission of malaria in Chennai.

## Abbreviations

OHT: overhead tank
RH: relative humidity
NVBDCP: National Vector Borne Disease Control Programme
UMS: urban malaria scheme
RBI: relative breeding index

## References

[1] Kumar V, Mangal A, Panesar S, Yadav G, Talwar R, Raut D (2014). Forecasting malaria cases using climatic factors in Delhi, India: a time series analysis. Malar Res Treat.

[2] Surya KS, Prajesh KT, Ashok KU, Mohammed AH, Agrawal OP (2014). Efficacy, human safety and collateral benefits of alphacypermethrin-treated long-lasting insecticidal net (Interceptor^®^) in a hyperendemic tribal area of Orissa, India. J Trop Dis.

[3] Malaria situation in India. nvbdcp.gov.in/Doc/mal_situation_Dec2014.pdf.

[4] Kumar DS, Andimuthu R, Rajan R, Venkatesan MS (2014). Spatial trend, environmental and socioeconomic factors associated with malaria prevalence in Chennai. Malar J.

[5] Shalini S, Chaudhuri S, Sutton PL, Mishra N, Srivastava N, David JK (2014). Chloroquine efficacy studies confirm drug susceptibility of *Plasmodium vivax* in Chennai, India. Malar J.

[6] Cator LJ, Thomas S, Paaijmans KP, Sangamithra R, Justin JA, Mathai MT (2013). Characterizing microclimate in urban malaria transmission settings: a case study from Chennai, India. Malar J.

[7] Guidelines of entomological surveillance of malaria vectors in Sri Lanka, Anti malaria campaign. 2009. http://www.malariacampaign.gov.lk/downloads/revised%20guidelines%20for%20entomological%20surveillance.pdf. Accessed 27 Jan 2016.

[8] WHO (2013). Malaria entomology and vector control—guide for participants.

[9] WHO (2013). Larval source management: a supplementary measure for malaria vector control: an operational manual.

[10] Ndenga BA, Simbauni JA, Mbugi JP, Githeko AK (2012). Physical, chemical and biological characteristics in habitats of high and low presence of Anopheline larvae in Western Kenya highlands. PLoS One.

[11] Kipyab PC, Khaemba BM, Mwangangi JM, Mbogo CM (2015). The physicochemical and environmental factors affecting the distribution of *Anopheles merus* along the Kenyan coast. Parasit Vectors.

[12] Nkondjio CA, Fossog BT, Ndo C, Djantio BM, Togouet SZ, Ambene PA (2011). *Anopheles gambiae* distribution and insecticide resistance in the cities of Douala and Yaoundé (Cameroon): influence of urban agriculture and pollution. Malar J.

[13] WHO (1997). Vector control: methods for use by individuals and communities.

[14] Das MK, Wattal S, Nanda N, Adak T (2004). Laboratory colonization of *Anopheles sundaicus*. Curr Sci.

[15] Nagpal BN, Sharma VP (1995). Indian anophelines.

[16] Nagpal BN, Srivastava A, Saxena R, Ansari MA, Dash AP, Das SC (2005). Pictorial identification key for Indian anophelines.

[17] WHO (2012). Training manual on malaria entomology.

[18] Bayoh MN, Lindsay SW (2003). Effect of temperature on the development of the aquatic stages of *Anopheles gambiae* sensu stricto (Diptera:Culicidae). Bull Entomol Res.

[19] Lyons CL, Coetzee M, Chown SL (2013). Stable and fluctuating temperature effects on the development rate and survival of two malaria vectors, *Anopheles arabiensis* and *Anopheles funestus*. Parasit Vectors.

[20] Impoinvil DE, Cardenas GA, Gihture JI, Mbogo CM, Beier JC (2007). Constant temperature and time period effects on *Anopheles gambiae* egg hatching. J Am Mosq Control Assoc.

[21] Huang J, Walker ED, Vulule J, Miller JR (2006). Daily temperature profiles in and around Western Kenyan larval habitats of *Anopheles gambiae* as related to egg mortality. Malar J.

[22] Imbahale SS, Paaijmans KP, Mukabana WR, Lammeren RV, Githeko AK, Takken W (2011). A longitudinal study on *Anopheles* mosquito larval abundance in distinct geographical and environmental settings in western Kenya. Malar J.

[23] Machault V, Gadiaga L, Vignolles C, Jarjaval F, Bouzid S, Sokhna C (2009). Highly focused anopheline breeding sites and malaria transmission in Dakar. Malar J.

[24] Kristan M, Abeku TA, Beard J, Okia M, Rapuoda B, Sang J (2008). Variations in entomological indices in relation to weather patterns and malaria incidence in East African highlands: implications for epidemic prevention and control. Malar J.

[25] Paaijmans KP, Wandago MO, Githeko AK, Takken W (2007). Unexpected high losses of *Anopheles gambiae* larvae due to rainfall. PLoS One.

[26] Yoshioka M, Couret J, Kim F, McMillan J, Burkot TR, Dotson EM (2012). Diet and density dependent competition affect larval performance and oviposition site selection in the mosquito species *Aedes albopictus* (Diptera:Culicidae). Parasit Vectors.

[27] Sumba LA, Ogbunugafor CB, Deng AL, Hassanali A (2008). Regulation of oviposition in *Anopheles gambiae s.s*: role of inter- and intra-specific signals. J Chem Ecol.

[28] Mwangangi JM, Muturi EJ, Shililu J, Muriu SM, Jacob B, Kabiru EW (2008). Contribution of different aquatic habitats to adult *Anopheles arabiensis* and *Culex quinquefasciatus* (Diptera:Culicidae) production in a rice agroecosystem in Mwea, Kenya. J Vector Ecol.

[29] Afify A, Galizia CG (2014). Gravid females of the mosquito *Aedes aegypti* avoid oviposition on *m*-cresol in the presence of the deterrent isomer *p*-cresol. Parasit Vectors.

[30] Suwonkerd W, Ritthison W, Ngo CT, Tainchum K, Bangs MJ, Chareonviriyaphap T (2013). Vector biology and malaria transmission in Southeast Asia, *Anopheles* mosquitoes—new insights into malaria vectors. InTech.

[31] Quraishi MS, Esghi N, Faghih MA (1966). Flight range, lengths of gonotrophic cycles, and longevity of P32-labeled *Anopheles stephensi* mysorensis. J Econ Entomol.

[32] Paaijmans KP, Heinig RL, Seliga RA, Blanford JI, Blanford SB, Murdock CC (2013). Temperature variations make ectotherms more sensitive to climate change. Glob Chang Biol.

[33] Mandal B, Biswas B, Banerjee A, Mukherjee TK, Nandi J (2011). Breeding propensity of *Anopheles stephensi* in chlorinated and rainwater containers in Kolkata City, India. J Vector Borne Dis.

[34] National Vector Borne Disease Control Programme, Directorate General of Health Services, Ministry of Health & Family Welfare, Malaria Situation in India (State-wise) from 2009 to 2013. http://nvbdcp.gov.in/malaria11.html. Accessed 1 July 2014.

[35] Polson KA, Brogdon WG, Rawlins SC, Chadee DD (2012). Impact of environmental temperatures on resistance to organophosphate insecticides in *Aedes aegypti* from Trinidad. Rev Panam Salud Publica.

[36] Yates WW (1950). Effect of temperature on the insecticidal action of mosquito larvicides. Mosq News.

[37] George L, Lenhart A, Toledo J, Lazaro A, Han WW, Velayudhan R, Runge Ranzinger S (2015). Community-effectiveness of temephos for dengue vector control: a systematic literature review. PLoS Negl Trop Dis.

[38] National Institute of Malaria Research (ICMR). Seven point action plan for malaria control in urban areas. MRC Technical Information Series No. 003/1996. p. 15.

